# Diffraction-based determination of single-crystal elastic constants of polycrystalline titanium alloys

**DOI:** 10.1107/S1600576719010720

**Published:** 2019-09-20

**Authors:** Alexander Heldmann, Markus Hoelzel, Michael Hofmann, Weimin Gan, Wolfgang W. Schmahl, Erika Griesshaber, Thomas Hansen, Norbert Schell, Winfried Petry

**Affiliations:** a Heinz Maier-Leibnitz Zentrum (MLZ), Technische Universität München, Lichtenbergstrasse 1, 85748 Garching, Germany; b German Engineering Materials Science Centre, Helmholtz-Zentrum Geesthacht, Max-Planck Strasse, 21502 Geesthacht, Germany; c Ludwig-Maximilians-Universität München, Department für Geo- und Umweltwissenschaften, Theresienstrasse 41, 80333 München, Germany; d Institut Laue–Langevin, 71 Avenue des Martyrs, 38042 Grenoble, France

**Keywords:** elastic constants, elasticity, stiffness, multiphase alloys

## Abstract

Single-crystal elastic constants have been derived by lattice strain measurements using neutron diffraction on polycrystalline Ti-6Al-4V, Ti-6Al-2Sn-4Zr-6Mo and Ti-3Al-8V-6Cr-4Zr-4Mo alloy samples. A variety of model approximations for the grain-to-grain interactions, namely approaches by Voigt, Reuss, Hill, Kroener, de Wit and Matthies, including texture weightings, have been applied and compared. A load-transfer approach for multiphase alloys was also implemented and the results are compared with single-phase data. For the materials under investigation, the results for multiphase alloys agree well with the results for single-phase materials in the corresponding phases. In this respect, all eight elastic constants in the dual-phase Ti-6Al-2Sn-4Zr-6Mo alloy have been derived for the first time.

## Abstract 2

The single-crystal elastic constants of polycrystalline titanium alloys have been determined using neutron and synchrotron powder diffraction.

## 1. Introduction   

Single-crystal elastic constants are essential material parameters in fundamental materials science as well as in engineering. In particular, the elastic properties of any poly­crystalline bulk material are based on its single-crystal elastic constants. A variety of micromechanical models have been established to describe the relations between the elastic properties of a polycrystalline bulk material and the single-crystal elastic constants, the most common applied approaches being those introduced by Voigt (1928 ▸), Reuss (1929 ▸), Hill (1952 ▸), Kroener (1958 ▸), de Wit (1997 ▸) and Matthies *et al.* (2001 ▸).

In classical stress analysis using diffraction, the residual stresses of alloys are determined by measuring the lattice strains for different sample orientations. Lattice strains and stresses are related by the diffraction elastic constants (DECs), which depend on the direction in the crystal as described by the lattice planes (*hkl*) of the corresponding Bragg reflections. DECs in turn are based on the single-crystal elastic constants. Thus, knowledge of the single-crystal elastic constants is essential for diffraction-based stress analysis.

The conventional stress analysis can be modified in such a way as to apply a defined external stress on the sample (by tensile load or compression) while the lattice strains are measured under various sample orientations. The single-crystal elastic constants of any polycrystalline material may then be determined by an inversion of the usual calculations applied in the residual stress analysis and fitting by a χ^2^-minimization technique. The feasibility of such an approach was demonstrated by Hauk & Kockelmann (1979 ▸) and later applied by various authors (Gnäupel-Herold *et al.*, 1998 ▸; Kisi & Howard, 1998 ▸; Singh *et al.*, 1998 ▸; Howard & Kisi, 1999 ▸; Matthies *et al.*, 2001 ▸; Fréour *et al.*, 2005 ▸; Stebner *et al.*, 2013 ▸; Lunt *et al.*, 2014 ▸; Kim *et al.*, 2016 ▸) for alloys and ceramics by diffraction under tensile stress or compression. This technique is of particular relevance for any engineering material which cannot be obtained as a single crystal, especially multiphase or highly twinned materials.

In this contribution, we will first review the background to this technique, with particular focus on the measurement geometry and data evaluation. For the validation of our data collection and evaluation procedure, face-centred cubic (f.c.c.) and body-centred cubic (b.c.c.) phases in steels and cast iron samples were studied. The results are compared with literature data on single-crystal elastic constants and bulk properties. Approaches for the modelling of grain-to-grain interactions by Voigt (1928 ▸), Reuss (1929 ▸), Hill (1952 ▸), Kroener (1958 ▸), de Wit (1997 ▸) and Matthies *et al.* (2001 ▸) are applied in the calculations. Since in earlier work the influence of the texture was not investigated in detail, we systematically examine here the influence of texture weightings compared with a quasi-isotropic approach. In addition, a load-partition model is included in the modelling approaches.

Despite the fact that titanium alloys are important high-performance alloys, few data are available concerning DECs or single-crystal elastic constants. Howard & Kisi (1999 ▸) obtained the single-crystal elastic constants in the near α-alloy Ti-6Al-4V by diffraction on a polycrystalline specimen. In a similar way, Fréour *et al.* (2005 ▸) determined the elastic constants of the b.c.c. phase in the dual-phase alloy Ti-17.

As the main part of this study, we present the results for the alloys Ti-6Al-4V (Ti64, near α-alloy), Ti-3Al-8V-6Cr-4Zr-4Mo (Ti38644, β-alloy) and Ti-6Al-2Sn-4Zr-6Mo (Ti6246, α- and β-alloy). The elastic constants of both phases in Ti6246 are compared with the results for the single-phase materials.

Ti64 is a high-strength titanium alloy and is considered as the ‘workhorse’ alloy for aerospace applications. It offers excellent strength and toughness up to 673 K combined with good fabricability. As a ‘near α-alloy’ it consists mainly of the hexagonal α phase and low amounts of a cubic β phase.

Ti6246 is a very high strength titanium alloy but with lower toughness and weldability than Ti64, although it can be operated at higher temperatures. It consists of two phases, one hexagonal α phase and one b.c.c. β phase.

In general, β-alloys have high corrosion resistance and very high strength but a lower elastic modulus than α-alloys.

## 2. Theory   

In the following, a general derivation of the current methods is provided. This includes the single-crystal elastic constants, their relation to the crystal lattice and their significance for the DECs. For multiphase alloys, a method of quantifying the load transfer from one phase to another as described by Behnken (2003 ▸) is implemented in the evaluation process. A fitting routine for the elastic constants based on the approach by Gnäupel-Herold *et al.* (1998 ▸) is applied to different micro-mechanical models. 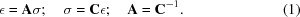
The well known Hooke’s law (1) provides the relation between the second-rank tensors of strains ∊ and stresses σ for any material under elastic strain. The proportional constants are given by the fourth-rank tensors of elastic compliances **A** and elastic constants **C**.

**Figure 1:**
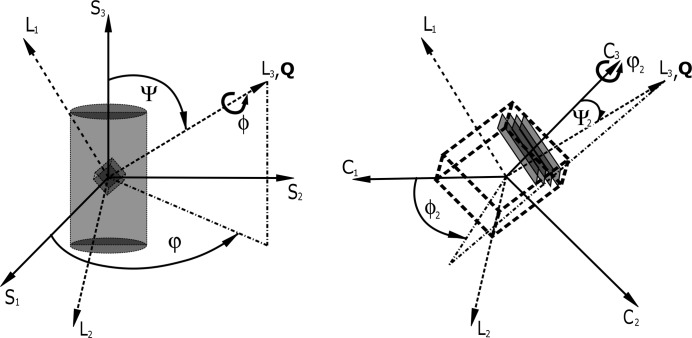
(Left) The different angles for transformation from the sample (*S*_*i*_, *i* = 1–3) to the measurement frame (*L*_*i*_, *i* = 1–3). (Right) The transformation from the crystal (*C*_*i*_, *i* = 1–3) to the measurement frame.

Diffraction studies under the influence of an applied mechanical load enable investigation of the strains perpendicular to particular (*hkl*) lattice planes, *i.e.* strains in different crystallographic directions. As outlined in detail by Gnäupel-Herold *et al.* (1998 ▸), one needs to consider the relations between three coordinate systems. The measurement frame is defined by the scattering vector **Q** coinciding with the reciprocal-lattice vector **h** for each lattice plane (*hkl*). The load axis determining the applied stress defines the sample frame, while the single-crystal elastic compliances are expressed in the crystal frame. As illustrated in Fig. 1 ▸, the measurement frame *L* is defined in such a way that the *L*_3_ axis is parallel to **Q**. In the sample frame *S*_3_ is oriented along the load axis in the case of uniaxial tensile (or compression) experiments. As shown in Fig. 1 ▸, the orientation between the scattering vector **Q** and the load axis (*S*_3_) is given by the angles ψ and ϕ. *S* can be transformed into *L* via the rotation ω as given in equation (2):
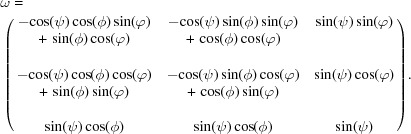


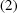
The transformation from the crystal frame into the measurement frame is done with the rotation ξ (Behnken, 2003 ▸):
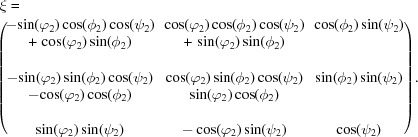


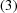
In order to ensure the correct orientations of *L*_3_ to the crystal frame, the rotations are defined by the Euler angles 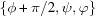 and 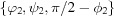 (Gnäupel-Herold *et al.*, 1998 ▸; Brakman, 1983 ▸).

From equation (1) the lattice strains in the measurement frame for a Bragg reflection (*hkl*) under orientations φ and ψ are given by
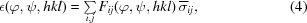
with the stress factor *F*_*ij*_ given by
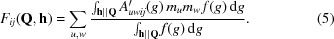


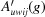
 are the general single-crystal compliances expressed in the measurement frame. Therefore **A** from equation (1) is transformed from the crystal into the measurement frame by the rotation matrix ξ in the following way: 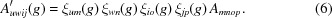
The function *f*(*g*) describes the orientation distribution of the grains (ODF) and is typically related to the sample frame (Behnken, 2003 ▸), *i.e.* in equation (5) the integrals are evaluated over all grains contributing to the diffraction signal.

In equation (4) the strains are measured along **Q**, and the only observed component of the strain tensor is ∊_33_. 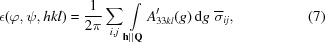


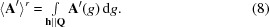
Equation (7) can be transformed with the knowledge of the rotation symmetry of the average tensor and the measurement direction in the measurement frame, yielding
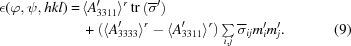
Equation (9) can be transformed into the sample reference frame, which leads to the general equation of stress analysis (Behnken, 2003 ▸) 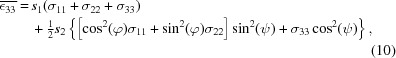
with the DECs *s*_1_(*hkl*) and 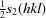


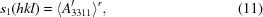


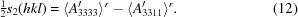
An analytical solution of DECs from the single-crystal elastic constants can be calculated for different crystal symmetries using a model assumption for the grain-to-grain interaction.

In order to establish relationships between bulk and single-crystalline properties, Voigt assumed that in all grains homogeneous strains appear as a function of an external stress. In this case the general elastic compliances are calculated from the averaged elastic constants 〈*c*(*g*)〉^−1^ (Voigt, 1928 ▸). Later, Reuss (1929 ▸) assumed homogeneous stress in grains; thus **A** depends on the elastic compliances. Kroener (1958 ▸) developed a model based on Eshelby’s assumption of spherical elastic inclusions in an isotropic matrix (Eshelby, 1957 ▸), which was extended by de Wit (1997 ▸). Thus,
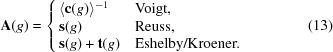
The DECs based on the Voigt (1928 ▸) model for any crystal symmetries are given in equations (14) and (15): 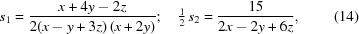


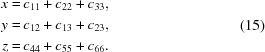
In equation (15) the single-crystal elastic constants are represented in Voigt’s notation.

The solutions for Reuss’s approximation have been developed by Behnken (2003 ▸) for arbitrary crystal symmetries: 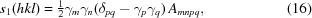


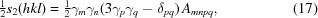


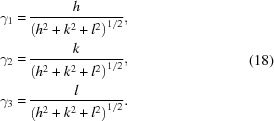
On the basis of these equations, one can derive the DECs for different crystal symmetries. In the special case of cubic crystal symmetry the DECs can written as
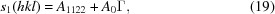


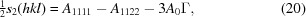
with *A*_0_ = *A*_1111_ − *A*_1122_ − 2*A*_1212_ and the crystal-orientation parameter Γ:
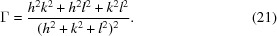
It has been shown by Hill (1952 ▸) that the assumptions made by Voigt (1928 ▸) and Reuss (1929 ▸) are only valid in specific cases, giving an upper and lower limit for the bulk elastic properties for most materials. Therefore, the arithmetic average of the bulk moduli obtained by both models was proposed for more realistic values. However, due to the tensor nature of the elastic constants, the physical relation **A** = **C** ^−1^ is not granted in this case. Matthies *et al.* (2001 ▸) showed that a geometric average between Voigt (1928 ▸) and Reuss (1929 ▸) calculations obeys the physical relation **A** = **C** ^−1^.

Kroener (1958 ▸) introduced a self-consistent model to solve the case of spherical inclusions in a matrix for cubic symmetries where the shear modulus *G* of the matrix is compared with the shear modulus *G*_0_ of the inclusion:
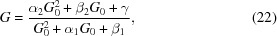
with the parameterization 
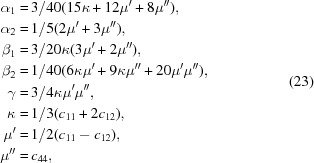
and DECs
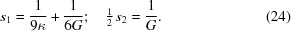



de Wit (1997 ▸) modified Kroener’s approach, recognizing that, by applying statistical averages, the stress is averaged over all spatial directions, whereas the strain is only measured in the direction normal to the diffraction planes. This leads to a slight modification in the description of the parameters (de Wit, 1997 ▸):
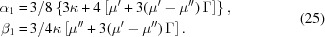



In a multi-phase alloy subjected to elastic strain, a load transfer towards stiffer phases is expected. Diffraction studies reveal the effective elastic constants of the individual phases due to their interactions with the other phases present. Behnken (2003 ▸) derived an approach to quantify the load transfer in multiphase systems with different elastic properties. The mean phase stress 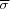 consists of a macro stress-dependent contribution and an independent part denoted by the index 0. 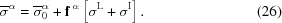


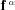
 is the fourth-rank tensor of the stress transition factors for phase α, σ^L^ is the applied load to the sample and σ^I^ are the residual stresses. To calculate the transition factors, the following equation is derived for an inclusion in a homogeneous matrix (Behnken, 2003 ▸):
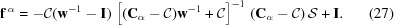
In the fourth-rank tensor equation (27), 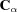 are the single-crystal elastic constants of the phase α (in this case the inclusions), **I** is the unity tensor, and 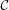 and 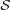 are the macroscopic elastic moduli and compliances, respectively, based on the following equation:
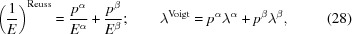
where the phase fractions of phases α and β are denoted by *p* ^α, β^, Young’s modulus is denoted by *E* and λ is a Lame constant. **w** ^−1^ depends on the shape of the inclusions and is often called the Eshelby tensor. An analytical solution has been found by Kneer (1965 ▸) for spherical and ellipsoidal inclusions:
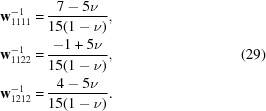
In the case of spherical inclusions, **w** ^−1^ is isotropic and depends only on the Poisson ratio ν (Kneer, 1965 ▸). The stresses of the matrix can be calculated either via the transition factors of the matrix which are obtained via 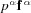 + 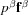 = **I** or via the condition 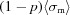 + 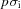 = 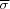, where the index m represents the matrix and i the inclusion, and *p* is the phase fraction of the inclusion.

One needs to address the minimization problem to obtain the single-crystal elastic constants from diffraction on polycrystalline materials. We choose the method of least squares (χ^2^ minimization) to optimize the elastic compliances by minimizing the sum of squares between deviations of measured and calculated observables.

The first possibility is to derive the DECs from the measured data and subsequently minimize the differences between them and the computed elastic constants (Gnäupel-Herold *et al.*, 1998 ▸),
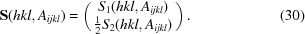
In equation (30), **S** is a two-dimensional quantity and the total length must be minimized. 
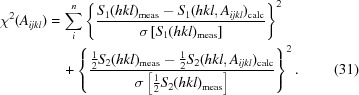
The technique for obtaining the values of *S*_1_(*hkl*)_meas_ and 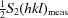 is explained in the following section. *S*_1_(*hkl*, *A*_*ijkl*_)_calc_ and 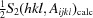 are computed with the help of equations (16), (18), (14) or (24), depending on the model used.

The second possibility is to express χ^2^ directly in terms of the measured strains 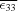, leading to the following expression for χ^2^: 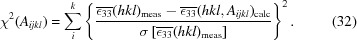
In principle, both techniques will lead to similar results, but there are important differences between them. Equation (31) is a more specialized form of equation (32), which leads to a large reduction in the summation terms of χ^2^, *e.g.* *k* ≫ *n* because the *S*_*i*_ are combined values from the different measurement directions and only depend on the scattering planes.

Additionally, in both cases the texture of the sample can be taken into account through weightings of either the DECs or the strains directly, based on the multiples of random distribution (m.r.d.) values from the ODF of the different scattering planes involved.

## 3. Experimental setup   

Diffraction studies were carried out using both neutron and high-energy X-ray diffractometers. The large gauge volume in neutron experiments yields higher grain statistics compared with X-ray studies. On the other hand, high-energy (short-wavelength) X-ray experiments enable the recording of complete Debye–Scherrer rings under a continuous increase of the applied load.

A load frame especially designed for elastic anisotropy and texture measurements was used, allowing the load axis to be oriented with respect to the incident beam by a χ rotation as on an Eulerian cradle (Hoelzel *et al.*, 2013 ▸).

**Figure 2:**
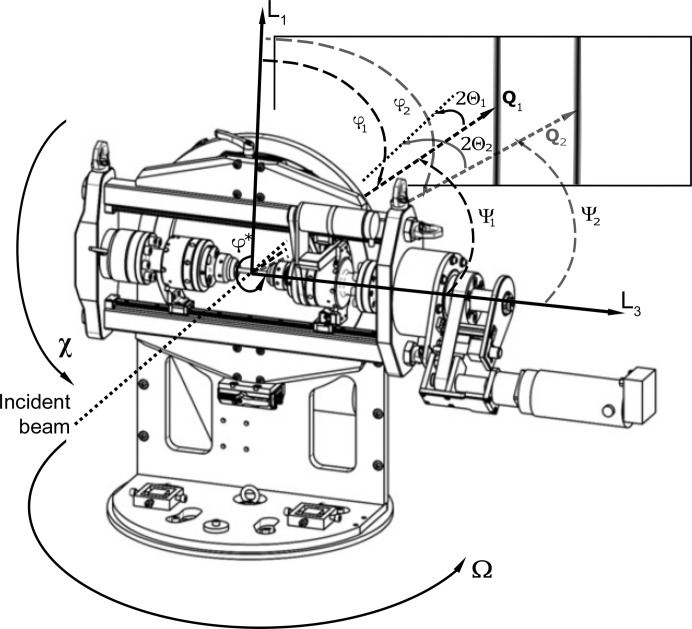
A schematic view of the rotatable tensile rig. The orientation between the sample and the incident beam is given by the angles Ω, χ and φ.

Neutron diffraction experiments were performed on the high-flux powder diffractometer D20 at the Institut Laue–Langevin in Grenoble, France (Hansen *et al.*, 2008 ▸) and the high-resolution powder diffractometer SPODI at the Heinz Maier-Leibnitz Zentrum (MLZ) in Garching near Munich, Germany (Hoelzel *et al.*, 2012 ▸). Both instruments operate at a constant wavelength and offer a broad scattering angle range up to 160° in 2Θ. In this setup the position of the sample frame with respect to the measurement frame is given by χ (tilt axis of the load frame) and Ω (rotation axis of the sample table), as shown in Fig. 2 ▸. This setup allows a simultaneous analysis of various (*hkl*) reflections, while the individual orientations between the scattering vectors **Q**(*hkl*) and the load axis need to be considered in the data analysis for each reflection individually. By changing the orientation of the load axis by χ and Ω, different angles ψ and φ can be obtained for each (*hkl*) reflection. In addition, measurements at different χ angles can be used to account for possible texture effects.

The experiments were done using standard tensile samples of 6 or 8 mm in diameter under uniaxial tension; no shear or bending forces were applied, nor did they occur on the sample during the tensile experiments. The macroscopic strain was measured along the load axis using a strain gauge (clip-on extensometer Sandner EXA-15). For each sample composition, tensile tests were carried out prior to the neutron diffraction studies to determine Young’s modulus and the yield stress. Diffraction patterns were collected at a minimum of three and up to five stress levels, up to a maximum value of about 70% of the yield stress. At each load step, measurements at a minimum of five and up to ten χ angles were performed.

To validate our method for data collection and analysis, ferritic (b.c.c.) and austentic (f.c.c) phases in steels and cast iron samples were studied on the SPODI diffractometer at a monochromator angle of 155° using Ge(551) for a wavelength of 1.5483 Å. The instrument offers a good angular resolution to separate the ferrite and austenite peaks and to observe their shifts under load at high precision. The investigations were carried out on ferritic structural steel S235JR, an austenitic stainless steel of AISI type 304 (X5CrNi 18-10), duplex steel (X2CrNiMoN 22-5-3) and an austempered ductile iron (ADI) sample consisting of ferrite, austenite and graphite.

The neutron scattering power of titanium alloys is quite poor. This is particularly the case for the β phase of titanium owing to the negative scattering length of Ti and a significant quantity of alloying elements (with positive scattering lengths). Therefore, the investigations on titanium alloys Ti-6Al-4V (Ti64, near α-alloy) and Ti-3Al-8V-6Cr-4Zr-4Mo (Ti38644, β-alloy) were performed on the D20 high-flux instrument at a monochromator angle of 90° using Ge(115) to achieve a wavelength of 1.544 Å. This setup offers a good angular resolution, sufficient for the analysis of strains. Samples of 8 mm diameter were measured under uniaxial tension at 0, 10, 20 and 30 kN.

X-ray diffraction experiments were carried out on Ti-6Al-2Sn-4Zr-6Mo on the High Energy Materials Science beamline (HEMS) at the synchrotron facility DESY in Hamburg, Germany (Schell *et al.*, 2014 ▸). An energy of 98.25 keV (corresponding to a wavelength of 0.12619 Å) was used to investigate tensile samples of 6 mm diameter. Since full Debye–Scherrer rings were obtained, no χ rotation was necessary to cover the different orientations of the load axis, which was kept perpendicular to the incident beam, *i.e.* Ω = 90°. The data were collected in 10 N steps up to 30 kN.

For texture analysis, neutron pole-figure measurements were carried out on the STRESS-SPEC instrument at the MLZ (Brokmeier *et al.*, 2011 ▸) using a Ge(311) monochromator for a wavelength of 1.68 Å. Bragg reflection intensities were measured from 0 to 360° in 5° steps in φ in seven different orientations in χ.

Preparation for electron backscatter diffraction (EBSD) and energy-dispersive X-ray diffraction (EDX) was performed by mechanical cutting and polishing. After cutting, plane grinding was performed with an MD-Mezzo surface followed by a single fine grinding with a 9 µm diamond suspension abrasive on an MD-Largo surface. The first polishing was performed with an MD-Chem surface and a 0.04 µm collodial silica abrasive and a final step of ion polishing with a Hitachi IM4000PLUS cross section ion polisher operated at 5 kV and 200 µA for 60 min with sample oscillation. The sample was cut along the cylinder axis, such that the sample normal is the transverse direction of the cylinder. EBSD maps were measured on a Hitachi SU5000 field-emission scanning electron microscope equipped with an Oxford Instruments NordlysII EBSD detector and an ULTIM MAX EDX detector. The employed acceleration voltage was 20 kV. EBDS and EDX signals were collected simultaneously with the sample inclined by 70° towards the EBSD detector. Data evaluation was performed with the Oxford Instruments *AZTec* and *CHANNEL5* software. The EDX analysis was carried out in standardless mode.

## 4. Data evaluation   

For the data evaluation, a software package was developed enabling the choice of all mentioned models and, optionally, the texture implementation. For dual-phase alloys, a possible load transfer from one phase to the other can be quantified.

**Figure 3:**
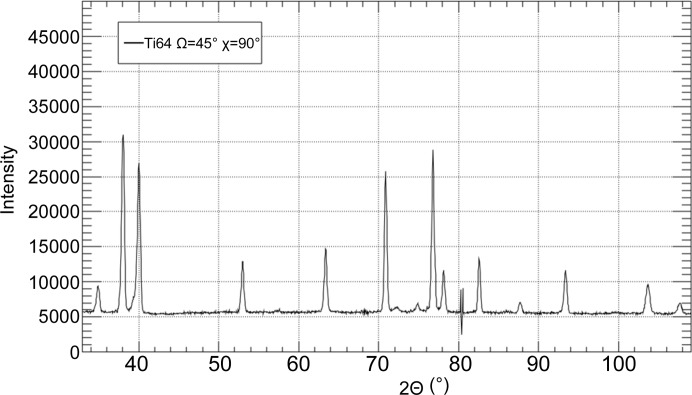
A diffraction pattern for Ti64 measured on D20 at Ω = 45° and χ = 90°. The artifact seen near 2Θ = 80° is caused by the detector and does not influence the results, since no *hkl* reflection is affected.

The *hkl*-dependent strains 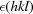 = 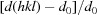 were obtained from the changes in the reflection positions under load in a set of diffraction patterns. An example diffraction pattern for Ti64 measured on D20 is shown in Fig. 3 ▸. To fit all free variables, at least three or five different Bragg peaks are needed for the cubic and hexagonal symmetries, respectively. The Bragg reflections were chosen to cover the maximum range of crystal directions to track the dependencies of the compliance tensor components.

For the ferritic phase (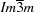) of the different iron and steel alloys, reflections {110}, {200}, {211}, {220} and {310} were evaluated, and for the austenitic phase (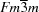) reflections {111}, {200}, {220}, {311} and {222}. For the hexagonal phase in the titanium alloys, all 16 reflections between {100} and {203} were evaluated, and for the cubic phase the {110}, {200}, {211}, {220} and {310} reflections were used for the evaluations.

**Figure 4:**
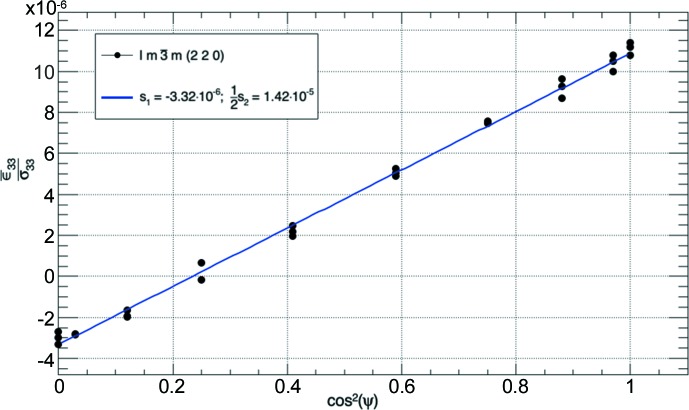
The linear relationship between the strain divided by the applied stress and cos^2^(ψ) is used to fit the DEC of the (220) plane of Ti38644.

In uniaxial tension or compression experiments only the σ_33_ component remains nonzero. In this case, equation (10) is simplified to
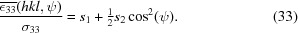
The equation shown above can be used to fit the DECs if the existing stresses are known or are given by an externally applied stress. The occurring strains for the lattice planes are derived from the shift of the corresponding Bragg peaks (*i.e.* relative changes in *d* spacing), while ψ is determined for every Bragg reflection separately by the Ω and χ orientations of the load axis of the tensile rig with respect to the measurement vector **Q**. An example fit of the (220) DEC of Ti38644 is given in Fig. 4 ▸.

It should be emphasized that this evaluation is not affected by intergranular residual stresses, as long as they do not change during the elastic loading, because the lattice strains are given by shifts of the reflections under load rather than by absolute *d* values.

**Figure 5:**
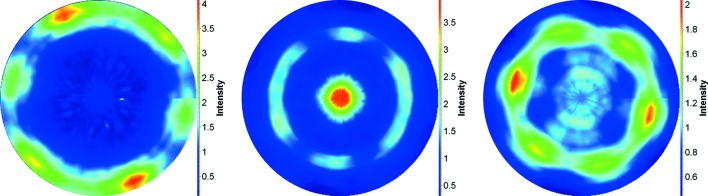
Pole figures of the Ti64 alloy. From left to right are shown the pole figures for the (002), (100) and (101) reflections, respectively.

The evaluation of the pole figures measured on STRESS-SPEC were done on the reflections {110} and {200} for 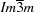, {111} and {200} for 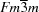, and {002}, {100}, {101}, {103}, {112} and {201} for the hexagonal phase. The pole figures of the first three planes are shown in Fig. 5 ▸.

The load transfer is calculated by a self-consistent scheme until the change in the transition factors after an iteration is smaller than a given limit. The initial values for the elastic constants are used in this approach, with no consideration of the load transfer. The stress factors for the inclusion are calculated with equation (27) defining the stress factors for the matrix. The parameters 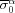 and σ^I^ in equation (26) are zero in our case, as the effects of residual stresses cancel out during the evaluation procedure as mentioned earlier:
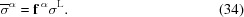
By means of equation (34) the associated stress for each Bragg reflection can be updated and the adjusted elastic constants are calculated. This iteration is repeated until the change in the stress factors is smaller than 10^−5^.

## 5. Results   

All obtained data were fitted with all available models except the Voigt (1928 ▸) model. The calculations based on the approach by Voigt showed large instabilities concerning the fitting routine, led to multiple results or did not converge. Therefore the results for Voigt (1928 ▸) are only shown once, for the structural steel S235JR.

The values for the single-phase alloys agree well with available literature data. The results for the ferritic phase are listed in Table 1 ▸. Finkel (2016 ▸) and Gnäupel-Herold *et al.* (1998 ▸) used Hill’s approach and obtained values of *c*_11_ = 232.0 GPa, *c*_12_ = 125.8 GPa and *c*_44_ = 115.2 GPa, and *c*_11_ = 224.9 GPa, *c*_12_ = 122.2 GPa and *c*_44_ = 120.7 GPa, respectively. Our results of *c*_11_ = 230.0 GPa, *c*_12_ = 121.0 GPa and *c*_44_ = 120.8 GPa using Hill’s approximation show good agreement with the literature data. Table 2 ▸ illustrates the results for the austenitic phase calculated for different models compared with the literature data. The data published by Ledbetter (1985 ▸) yielding *c*_11_ = 209.0 GPa, *c*_12_ = 133.0 GPa and *c*_44_ = 121.0 GPa were predicted on the basis of Kroener’s model. Comparison with our values for Kroener’s model of 208.0, 135.7 and 116.3 GPa for the isotropic approximation and 204.8, 138.9 and 124.2 GPa for the texture adaptation shows good agreement in both cases.

The results for the duplex steel and ADI are shown in Tables 3 ▸ and 4 ▸, respectively. Both consist of austenitic and ferritic phases. However, ADI contains a large amount of graphite of approximately 10 vol.% in the form of nodules with Young’s and shear moduli of essentially zero. For *c*_11_ and *c*_12_ the deviation is of the same order of magnitude as the uncertainty in the values, but for *c*_44_ the observed deviation is higher. In tensile experiments the parameter *c*_44_ is related to the shear stresses/strains and is therefore only indirectly accessible with the different orientations covered during the measurements. This leads to higher uncertainties for *c*_44_ during the determination in the fitting process. The accuracy for *c*_44_ may be improved by including torsion experiments, as suggested by Woracek *et al.* (2012 ▸).

The tables also reveal larger discrepancies between the models in the Zener anisotropy defined by *A* = 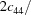 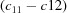 (Zener, 1948 ▸) and the 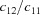 ratio. In a systematic study of the single-crystal elastic constants of different monocrystals of austenitic stainless steels and Fe–Cr–Ni alloys, Ledbetter (1985 ▸) found that both ratios remain nearly constant at *A* = 3.53 and 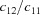 = 0.635 (Ledbetter, 1985 ▸). Owing to the low variance in the values of *A* and 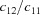 in Ledbetter’s study, we believe these ratios can be used to estimate the best model for the material under investigation.

In all models it turns out that the texture does not influence the results above their uncertainties. This is most likely due to the elastic measurements being performed on single-crystal domains and therefore not directly affected by the texture. The only parameter affected is the average strain measured via diffraction. Matthies *et al.* (2001 ▸) concluded that just a couple of thousand grains are enough to ensure the statistical significance of the average strains. Therefore, the isotropic grain orientation assumption is considered to be adequate for the investigated materials as the texture is assumed not to change significantly in the elastic regime with the applied load. Further, our diffraction data show no changes in the Bragg intensities in the elastic regime.

The elastic properties of both phases in duplex steel are very similar, resulting in a load transfer of about 0.3%. Thus, the load transfer approach reveals practically the same elastic constants. A similar behaviour would be expected for ADI but was not investigated further here as the additional graphite phase could not be taken into account.

**Figure 6:**
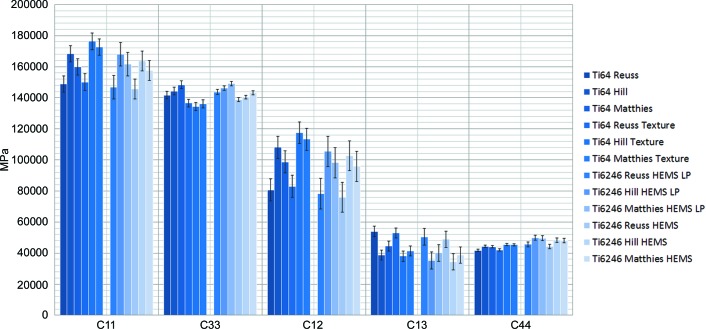
An overview of the single-crystal elastic constants of the h.c.p. phase in Ti64 and Ti6246.

**Figure 7:**
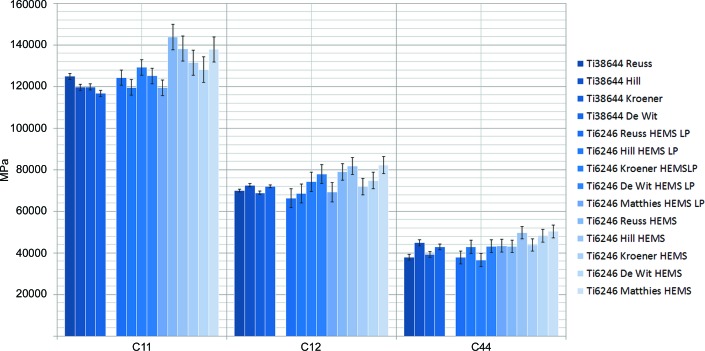
An overview of the single-crystal elastic constants of the b.c.c. phase in Ti38644 and Ti6246.

The results for the obtained elastic constants in the hexagonal α phase in Ti64 and Ti6246 are shown in Fig. 6 ▸, while Fig. 7 ▸ illustrates the corresponding values for the β phase in Ti38644 and Ti6246. To the best of our knowledge, the analysis of Ti6246 is the first example of deriving all elastic constants in a dual-phase (α + β) titanium alloy. Tables 5 ▸, 6 ▸ and 7 ▸ reveal quite similar results for the different model assumptions. In addition, the values obtained for the α + β alloy Ti6246 agree quite well, especially if the load transfer is taken into account, with the corresponding data for the α phase in Ti64 and the β phase in Ti38644, respectively. Table 6 ▸ also includes the results of Howard & Kisi (1999 ▸) on the Ti64 alloy determined by the Reuss (1929 ▸) approach, as well as values obtained by ultrasonic studies on single crystals of pure Ti (Fisher & Renken, 1964 ▸).

Good agreement with ultrasonic data was found in the hexagonal phases for *c*_11_ and *c*_44_. The largest deviation between our results and earlier work is found for *c*_33_, and minor deviations in the *c*_12_ and *c*_13_ elastic constants. In hexagonal systems the behaviour in the *ab* plane is completely isotropic owing to the requirement for the 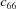 = 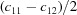 elastic constant. As we are able to determine *c*_11_ and *c*_12_ accurately, this also gives access to the shearing parameter *c*_66_. On the other hand, hexagonal systems show additional anisotropies regarding the *c* axis which influence the precision of parameters *c*_12_ and *c*_13_.

Macroscopic Young’s moduli were measured in a series of tensile tests to 106.6 (2.1) GPa for Ti64, to 88.5 (1.6) GPa for Ti38644 and to 113.6 (2.6) GPa for Ti6246. Tables 5 ▸, 6 ▸ and 7 ▸ indicate that there is very good agreement for Ti64 and Ti38644 between these and the values calculated on the basis of the diffraction studies, while somewhat higher values were obtained for Ti6246.

**Figure 8:**
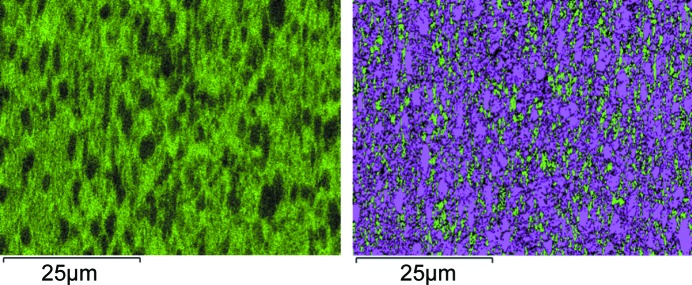
EDX and EBSD results for the Ti6246 alloy. (Left) The molybdenum distribution (0–9 wt%). (Right) The distribution of cubic (green) and hexagonal (purple) phases. Black pixels on the EBSD are not indexable.

The phase fractions of the dual-phase Ti6246 alloys were evaluated from the obtained diffraction patterns using Rietveld analysis (*MAUD* and *FullProf* suite) (Lutterotti *et al.*, 1997 ▸; Rodriguez-Carvajal, 1993 ▸). For the alloy, phase fractions of 78 and 22% were found for the α and β phases, respectively. The results of phase distribution versus phase composition obtained by EBSD and EDX are shown for the Ti6246 alloy in Fig. 8 ▸. The phase fractions determined by EBSD amount to 78 and 22%, the same as for the Rietveld analysis. The main chemical difference between the two phases resides in the Mo content and the compositions are tabulated in Table 8 ▸. While the doping elements Sn, Zr and Al are relatively homogeneously distributed, Mo shows an inhomogeneous distribution with elliptical Mo-poor regions in the 5–10 µm range (Fig. 8 ▸).

The load partitioning applied to the Ti6246 sample indicates a significant load transfer from the β phase to the α phase. The stress in the β phase is reduced by 11.01% and transferred to the α phase, increasing its stress by 3.10% (note that the smaller increase is mainly due to the ratio of phase fractions of around 1:4). The single-crystal elastic constants corrected for load transfer are shown in Table 7 ▸. They reveal a clear shift from the uncorrected values towards the corresponding values of the single-phase samples. The impact of the load transfer in the β phase for the Reuss (1929 ▸), Hill (1952 ▸) and Matthies *et al.* (2001 ▸) models yields a change of about 10% in most cases, whereas for the Kroener (1958 ▸) and de Wit (1997 ▸) models the changes in the elastic constants remain below 5%. *c*_11_ of the β phase was shifted from 143.8 to 124.3 GPa, *c*_12_ from 78.9 to 66.3 GPa and *c*_44_ from 43.1 to 37.8 GPa for the Reuss model. The single-phase values of 124.9, 69.9 and 38.0 GPa for the same model almost match the ‘load-transfer-corrected’ ones. Similar behaviour is also observed for all other models. Owing to the lower increase in stress in the α phase the changes remain rather small, but the results achieved with the load-transfer model match the single-phase values more consistently. The comparatively small changes in the elastic constants when using the Kroener (1958 ▸) and de Wit (1997 ▸) models are supposedly due to the assumption of inclusions in a matrix, which is essentially the same assumption as used for the load transfer.

Similar elastic constants for α phases in different titanium alloys can be expected owing to the low quantity of alloying elements, shown already by the comparison of Howard & Kisi (1999 ▸) with pure titanium. Significant variations in the elastic constants for β phases in different alloys can be found in the literature (Hounkpati *et al.*, 2016 ▸; Fréour *et al.*, 2005 ▸). This may result from the fact that β-alloys contain a large number of different β-stabilizing elements in contrast with the α phase. However, our investigations show a larger impact of the modelling between the two investigated β phases.

## 6. Conclusions   

Diffraction experiments are a feasible and powerful route to determine single-crystal elastic constants from polycrystalline multiphase alloys due to the phase selectivity, as shown in the case of Ti6246.

The data-evaluation procedure included several approximations to consider grain-to-grain interactions in polycrystals based on the approches by Voigt (1928 ▸), Reuss (1929 ▸), Hill (1952 ▸), Kroener (1958 ▸), de Wit (1997 ▸) and Matthies *et al.* (2001 ▸). In terms of computational stability, the Reuss (1929 ▸), Hill (1952 ▸) and Matthies *et al.* (2001 ▸) assumptions were the most stable in our data analysis. Concerning the approaches of Kroener (1958 ▸) and de Wit (1997 ▸), not all possible combinations of the elastic constants led to a solution of the self-consistent equation (22). The Voigt equations could only be used for the evaluation with very limiting constraints. Good indicators of which models will suit best are the anisotropy values and the *c*_12_/*c*_11_ ratios obtained for the different models. We systematically investigated the effects of the texture on the modelling and find its influence to be smaller than the differences observed for different grain-to-grain interaction models.

For the first time a full analysis of the load transfer has been included in the evaluation of the elastic constants from diffraction data for the example of Ti6246.

For the dual-phase alloy Ti6246, the load-transfer approach allows a direct comparison of measured ‘effective’ elastic constants with ‘load-transfer-corrected’ elastic constants. In this alloy, a significant load relocation of about 14% was found from the β to the α phase. By including the load transfer, the evaluations of the constants of the β phase in the dual-phase alloy were significantly shifted towards those of the single-phase alloys, while the effect of the load transfer on the α phase was significantly smaller. In particular, the load-transfer-corrected elastic constants in the α phase of Ti6246 show good agreement with the corresponding values for the near α-alloy in Ti64. In addition, for the β phase the load-transfer-corrected constants are in excellent agreement with the corresponding single-phase result for the pure β-alloy.

## Figures and Tables

**Table 1:** The single-crystal elastic constants and bulk properties for S235JR measured on SPODI The anisotropy *A* is calculated after Zener (1948 ▸). For the Voigt model, the fitting routine does not converge to a single solution.

Model	*c*_11_ (GPa)	*c*_12_ (GPa)	*c*_44_ (GPa)	*E* (GPa)	*G* (GPa)	μ (TPa^−1^)	ν	*A*	*c*_12_/*c*_11_
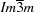									
Voigt	199	142	90	235	82	−12.10	0.436	3.1	0.71
Reuss	240	118	106	214	84	−7.51	0.276	1.7	0.49
Hill†	230	121	121	210	82	−7.57	0.278	2.2	0.53
Kroener	229	129	109	207	80	−7.86	0.288	2.2	0.56
de Wit	184	89	120	203	84	−5.51	0.219	2.5	0.49
Matthies†	224	129	112	216	85	−7.24	0.27	2.5	0.57
Finkel (2016 ▸)	232	126	115	220	86	−8.20	0.289	2.2	0.54
Gnäupel-Herold *et al.* (1998 ▸)	225	122	121	217	86	−8.05	0.283	2.4	0.54
Adams *et al.* (2006 ▸)	240	136	121					2.3	0.60
Kim & Johnson (2007 ▸)	232	135	116	212	82		0.289	2.4	0.58

† The anisotropy was fixed for these fits.

**Table 2:** The results for AISI type 304 (X5CrNi 18-10), a single-phase stainless steel, measured on SPODI; the texture data were measured on STRESS-SPEC The anisotropy *A* is calculated after Zener (1948 ▸).

Model	*c*_11_ (GPa)	*c*_12_ (GPa)	*c*_44_ (GPa)	*E* (GPa)	*G* (GPa)	μ (TPa^−1^)	ν	*A*	*c*_12_/*c*_11_
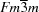									
Reuss	226	108	100	202	80	−7.85	0.271	1.7	0.48
Reuss†	225	107	100	202	80	−7.77	0.269	1.8	0.47
Hill	210	115	138	202	80	−7.85	0.271	2.9	0.55
Hill†	209	114	140	202	80	−7.77	0.269	3.0	0.55
Kroener	208	136	116	192	74	−8.97	0.300	3.2	0.65
Kroener†	205	139	124	194	75	−8.89	0.299	3.8	0.68
de Wit	212	130	121	204	80	−8.30	0.284	2.9	0.61
de Wit†	212	130	122	204	80	−8.27	0.284	3.0	0.61
Matthies	214	121	135	202	80	−7.85	0.271	2.9	0.56
Matthies†	213	120	136	202	80	−7.80	0.270	2.9	0.56
Finkel (2016 ▸)	207	121	119	202	79	−9.76	0.296	2.8	0.59
Ledbetter (1985 ▸)	209	133	121	197	76	−8.27	0.290	3.2	0.64
Ledbetter (1984 ▸)	205	138	126					3.8	0.67

† For the fitting of the elastic constants the DEC texture adaptation was used.

**Table 3:** The single-crystal elastic constants for X2CrNiMoN 22-5-3 duplex steel, the dual-phase stainless steel alloy, measured on SPODI; the texture data were measured on STRESS-SPEC The anisotropy *A* is calculated after Zener (1948 ▸).

Model	*c*_11_ (GPa)	*c*_12_ (GPa)	*c*_44_ (GPa)	*E* (GPa)	*G* (GPa)	μ (TPa^−1^)	ν	*A*	*c*_12_/*c*_11_
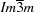									
Reuss	227	121	70	165	63	−11.7	0.323	1.3	0.53
Reuss†	228	121	70	165	62	−11.8	0.324	1.3	0.53
Hill	218	125	81	165	63	−11.7	0.323	1.8	0.58
Hill†	219	125	79	165	62	−11.8	0.324	1.7	0.57
Kroener	221	126	76	167	63	−11.5	0.324	1.6	0.57
Kroener†	210	137	87	164	62	−12.1	0.331	2.4	0.66
de Wit	214	126	79	165	63	−11.7	0.323	1.8	0.59
de Wit†	206	117	78	164	62	−11.4	0.314	1.8	0.57
Matthies	217	126	82	166	63	−11.7	0.322	1.8	0.58
Matthies†	219	125	79	165	63	−11.8	0.324	1.7	0.58
Finkel (2016 ▸)	210	108	83	177	69	−9.9	0.296	1.6	0.51
Kim *et al.* (2016 ▸)	222	144	114	194	74	−11.8	0.317	2.9	0.65
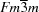									
Reuss	203	103	89	177	70	−9.62	0.284	1.8	0.51
Reuss†	208	107	88	178	70	−9.75	0.288	1.7	0.51
Hill	189	110	129	177	70	−9.62	0.284	3.3	0.58
Hill†	194	114	125	178	70	−9.75	0.288	3.1	0.58
Kroener	198	109	105	190	75	−7.85	0.272	2.4	0.55
Kroener†	189	120	120	190	74	−8.17	0.279	3.5	0.63
de Wit	222	154	112	186	71	−1.10	0.324	3.3	0.70
de Wit†	221	156	110	183	69	−11.40	0.328	3.4	0.70
Matthies	197	117	119	178	70	−9.66	0.286	3.0	0.59
Matthies†	201	119	116	179	70	−9.74	0.290	2.8	0.59
Finkel (2016 ▸)	189	110	125	198	79	−9.61	0.285	3.2	0.58
Kim *et al.* (2016 ▸)	207	134	114	188	72	−12.29	0.311	3.1	0.65

† For the fitting of the elastic constants the DEC texture adaptation was used.

**Table 4:** The single-crystal elastic constants for austempered ductile iron consisting of ferrite, austenite and graphite measured on SPODI; the texture data were measured on STRESS-SPEC The anisotropy *A* is calculated after Zener (1948 ▸).

Model	*c*_11_ (GPa)	*c*_12_ (GPa)	*c*_44_ (GPa)	*E* (GPa)	*G* (GPa)	μ (TPa^−1^)	ν	*A*	*c*_12_/*c*_11_
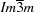									
Reuss	232	107	88	195	76	−8.24	0.281	1.4	0.46
Reuss†	218	103	92	191	75	−8.23	0.274	1.6	0.47
Hill	220	113	104	76	195	−8.24	0.281	2.0	0.51
Hill†	204	110	120	191	75	−8.23	0.274	2.6	0.54
Kroener	228	104	90	197	77	−7.67	0.274	1.5	0.46
Kroener†	231	90	101	200	79	−7.24	0.266	1.4	0.44
de Wit	217	116	102	198	78	−8.00	0.279	2.0	0.53
de Wit†	216	115	102	198	78	−7.98	0.278	2.0	0.53
Matthies	220	114	105	195	76	−8.16	0.280	2.0	0.52
Matthies†	202	126	112	192	76	−8.06	0.270	2.8	0.56
Finkel (2016 ▸)	201	124	108	185	72			2.8	0.62
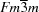									
Reuss	203	92	85	179	71	−8.59	0.269	1.6	0.46
Reuss†	204	94	85	179	71	−8.73	0.272	1.5	0.46
Hill	190	99	109	179	71	−8.59	0.269	2.4	0.52
Hill†	191	100	108	179	71	−8.73	0.272	2.4	0.52
Kroener	200	88	85	182	72	7.65	0.259	1.5	0.44
Kroener†	205	91	84	182	72	−7.93	0.266	1.5	0.45
de Wit	184	105	103	179	71	−8.84	0.273	2.6	0.57
de Wit†	183	104	103	178	70	−8.82	0.272	2.6	0.57
Matthies	193	101	105	179	71	−8.48	0.268	2.3	0.52
Matthies†	195	102	104	179	71	−8.62	0.271	2.3	0.53
Finkel (2016 ▸)	192	102	96	180	71			2.1	0.53

† For the fitting of the elastic constants the DEC texture adaptation was used.

**Table 5:** The single-crystal elastic constants and macroscopic values for Ti38644 measured on D20 The anisotropy *A* is calculated after Zener (1948 ▸).

Model	*c*_11_ (GPa)	*c*_12_ (GPa)	*c*_44_ (GPa)	*E* (GPa)	*G* (GPa)	μ (TPa^−1^)	ν	*A*	*c*_12_/*c*_11_
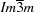									
Reuss	125	70	38	86	32	−23.91	0.337	1.4	0.56
Hill	120	73	45	86	32	−23.91	0.337	1.9	0.61
Kroener	120	69	88	39	33	−22.39	0.329	1.5	0.58
de Wit	117	72	43	32	−23.63	0.334	86	1.9	0.62
Matthies	120	73	45	86	33	−23.76	0.336	2.0	0.61

**Table 6:** The single-crystal elastic constants for Ti64 measured on D20; texture data were measured on STRESS-SPEC

Model	*c*_11_ (GPa)	*c*_33_ (GPa)	*c*_12_ (GPa)	*c*_13_ (GPa)	*c*_44_ (GPa)	*E* (GPa)	*G* (GPa)	μ (TPa^−1^)	ν
*P*6(3)/*mmc*									
Reuss	149	142	81	54	42	104	40	−16.9	0.303
Reuss†	150	137	83	53	42	103	40	−17.0	0.302
Hill	168	144	108	39	44	104	40	−16.9	0.303
Hill†	176	134	118	38	46	103	40	−17.0	0.302
Matthies	160	148	99	45	44	104	40	−16.9	0.302
Matthies†	173	136	113	41	45	103	40	−16.9	0.299
Howard & Kisi (1999 ▸)	154	173	82	61	45	114	43	−15.9	0.307
Fisher & Renken (1964 ▸)	162	181	92	69	47	116	44	−16.6	0.319

† For the fitting of the elastic constants the DEC texture adaptation was used.

**Table 7:** The single-crystal elastic constants for Ti6246, which consists of a hexagonal α phase and a b.c.c. β phase, measured on P07 HEMS

Model	*c*_11_ (GPa)	*c*_33_ (GPa)	*c*_12_ (GPa)	*c*_13_ (GPa)	*c*_44_ (GPa)	*E* (GPa)	*G* (GPa)	μ (TPa^−1^)	ν
*P*6(3)/*mmc*									
Reuss	146	139	76	49	43	103	39	−17.7	0.311
Reuss†	150	144	78	51	46	106	41	−17.2	0.311
Hill	164	140	103	35	48	103	39	−17.7	0.311
Hill†	168	146	105	35	50	106	41	−17.2	0.311
Matthies	157	143	96	39	48	103	39	−17.5	0.309
Matthies†	162	149	98	40	50	106	41	−17.0	0.309
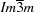									
Reuss	144		79		43	99	37	−20.5	0.335
Reuss†	124		66		38	88	33	−22.6	0.329
Hill‡	138		82		50	99	37	−20.5	0.335
Hill†	120		69		43	88	33	−22.6	0.329
Kroener	132		72		44	99	38	−19.0	0.320
Kroener†	129		74		37	88	32	−23.7	0.342
de Wit	128		75		48	99	37	−19.6	0.323
de Wit†	125		78		43	91	34	−22.6	0.338
Matthies‡	138		81		50	100	37	−20.4	0.335
Matthies†	119		69		43	88	33	−22.6	0.329

† Calculated with load partitioning.

‡ The anisotropy was fixed for these fits.

**Table 8:** Composition (wt%) of Ti6246 measured with EDX The results are presented for the Mo-poor and Mo-rich regions, as well as the average overall composition.

Mo-poor regions (hexagonal α phase)		Mo-rich regions (cubic β phase)		Overall average composition	
Ti	83.0–87.5	Ti	78.7–80.0	Ti	82.87
Mo	2.0–2.8	Mo	6.6–8.3	Mo	5.84
Al	5.6–6.5	Al	4.6–5.1	Al	5.39
Zr	3.5–3.6	Zr	3.9–4.0	Zr	3.84
Sn	1.8–2.4	Sn	1.9–2.1	Sn	2.06

## References

[bb1] Adams, J. J., Agosta, D. S., Leisure, R. G. & Ledbetter, H. M. (2006). *J. Appl. Phys.* **100**, 113530.

[bb2] Behnken, H. (2003). *Mikrospannungen in vielkristallinen und heterogenen Werkstoffen.* Herzogenrath: Shaker Verlag.

[bb3] Brakman, C. M. (1983). *J. Appl. Cryst.* **16**, 325–340.

[bb4] Brokmeier, H. G., Gan, W. M., Randau, C., Völler, M., Rebelo-Kornmeier, J. & Hofmann, M. (2011). *Nucl. Instrum. Methods Phys. Res. A*, **642**, 87–92.

[bb6] Eshelby, J. D. (1957). *Proc. R. Soc. London Ser. A*, **241**, 376–396.

[bb7] Finkel, M. (2016). Master’s thesis, Technische Universität München, Germany.

[bb8] Fisher, E. S. & Renken, C. J. (1964). *Phys. Rev.* **135**, A482–A494.

[bb9] Fréour, S., Gloaguen, D., François, M., Perronnet, A. & Guillén, R. (2005). *J. Appl. Cryst.* **38**, 30–37.

[bb10] Gnäupel-Herold, T., Brand, P. C. & Prask, H. J. (1998). *J. Appl. Cryst.* **31**, 929–935.

[bb11] Hansen, T. C., Henry, P. F., Fischer, H. E., Torregrossa, J. & Convert, P. (2008). *Meas. Sci. Technol.* **19**, 034001.

[bb12] Hauk, V. & Kockelmann, H. (1979). *Z. Metallkd.* **70**, 500–502.

[bb13] Hill, R. (1952). *Proc. Phys. Soc. A*, **65**, 349–354.

[bb14] Hoelzel, M., Gan, W. M., Hofmann, M., Randau, C., Seidl, G., Jüttner, P. & Schmahl, W. W. (2013). *Nucl. Instrum. Methods Phys. Res. A*, **711**, 101–105.

[bb15] Hoelzel, M., Senyshyn, A., Juenke, N., Boysen, H., Schmahl, W. W. & Fuess, H. (2012). *Nucl. Instrum. Methods Phys. Res. A*, **667**, 32–37.

[bb16] Hounkpati, V., Fréour, S., Gloaguen, D., Legrand, V., Kelleher, J., Kockelmann, W. & Kabra, S. (2016). *Acta Mater.* **109**, 341–352.

[bb17] Howard, C. J. & Kisi, E. H. (1999). *J. Appl. Cryst.* **32**, 624–633.

[bb18] Kim, S. A. & Johnson, W. L. (2007). *Mater. Sci. Eng. A*, **452–453**, 633–639.

[bb19] Kim, Y., Kim, Y. M., Koh, J.-Y., Lee, T.-H., Woo, W. C. & Han, H. N. (2016). *Scr. Mater.* **119**, 1–4.

[bb20] Kisi, E. H. & Howard, C. J. (1998). *J. Am. Ceram. Soc.* **81**, 1682–1684.

[bb21] Kneer, G. (1965). *Phys. Status Solidi B*, **9**, 825–838.

[bb22] Kroener, E. (1958). *Z. Phys.* **151**, 504–518.

[bb23] Ledbetter, H. M. (1984). *Phys. Status Solidi A*, **85**, 89–96.

[bb24] Ledbetter, H. M. (1985). *Phys. B+C*, **128**, 1–4.

[bb25] Lunt, A. J. G., Xie, M. Y., Baimpas, N., Zhang, S. Y., Kabra, S., Kelleher, J., Neo, T. K. & Korsunsky, A. M. (2014). *J. Appl. Phys.* **116**, 053509.

[bb26] Lutterotti, L., Matthies, S., Wenk, H. -R. S., Schultz, A. & Richardson, J. W. (1997). *J. Appl. Phys.* **81**, 594–600.

[bb27] Matthies, S., Priesmeyer, H. G. & Daymond, M. R. (2001). *J. Appl. Cryst.* **34**, 585–601.

[bb28] Reuss, A. (1929). *Z. Angew. Math. Mech.* **9**, 49–58.

[bb29] Rodríguez-Carvajal, J. (1993). *Physica B*, **192**, 55–69.

[bb30] Schell, N., King, A., Beckmann, F., Fischer, T., Müller, M. & Schreyer, A. (2014). *Mater. Sci. Forum*, **772**, 57–61.

[bb31] Singh, A. K., Mao, H.-K., Shu, J. & Hemley, R. J. (1998). *Phys. Rev. Lett.* **80**, 2157–2160.

[bb32] Stebner, A. P., Brown, D. W. & Brinson, L. C. (2013). *Appl. Phys. Lett.* **102**, 211908.

[bb33] Voigt, W. (1928). *Lehrbuch der Kristallphysik.* Stuttgart: Teubner Verlag.

[bb5] Wit, R. de (1997). *J. Appl. Cryst.* **30**, 510–511.

[bb34] Woracek, R., Bunn, J. R., Penumadu, D. & Hubbard, C. R. (2012). *Appl. Phys. Lett.* **100**, 191904.

[bb35] Zener, C. (1948). *Elasticity and Anelasticity of Metals.* University of Chicago Press.

